# What are the assets and weaknesses of HFO detectors? A benchmark framework based on realistic simulations

**DOI:** 10.1371/journal.pone.0174702

**Published:** 2017-04-13

**Authors:** Nicolas Roehri, Francesca Pizzo, Fabrice Bartolomei, Fabrice Wendling, Christian-George Bénar

**Affiliations:** 1 Aix Marseille Univ, Inserm, INS, Institut de Neurosciences des Systèmes, Marseille, France; 2 APHM, Timone hospital, Clinical Neurophysiology, Marseille, France; 3 INSERM U1099, Université de Rennes 1, LTSI, Rennes, France; McGill University, CANADA

## Abstract

High-frequency oscillations (HFO) have been suggested as biomarkers of epileptic tissues. While visual marking of these short and small oscillations is tedious and time-consuming, automatic HFO detectors have not yet met a large consensus. Even though detectors have been shown to perform well when validated against visual marking, the large number of false detections due to their lack of robustness hinder their clinical application. In this study, we developed a validation framework based on realistic and controlled simulations to quantify precisely the assets and weaknesses of current detectors. We constructed a dictionary of synthesized elements—HFOs and epileptic spikes—from different patients and brain areas by extracting these elements from the original data using discrete wavelet transform coefficients. These elements were then added to their corresponding simulated background activity (preserving patient- and region- specific spectra). We tested five existing detectors against this benchmark. Compared to other studies confronting detectors, we did not only ranked them according their performance but we investigated the reasons leading to these results. Our simulations, thanks to their realism and their variability, enabled us to highlight unreported issues of current detectors: (1) the lack of robust estimation of the background activity, (2) the underestimated impact of the 1/*f* spectrum, and (3) the inadequate criteria defining an HFO. We believe that our benchmark framework could be a valuable tool to translate HFOs into a clinical environment.

## Introduction

High-frequency oscillations (HFOs) are putative markers of epileptogenicity [1, 2]. The visual review of HFOs on intracerebral electroencephalography is time-consuming and tedious, and suffers from poor inter-reviewer reliability [3]. There is thus a crucial need for automatic detection in order to translate HFOs into clinical practice. Many different HFO detectors have been designed [4–10] but none has yet met a large consensus. The validation of the detectors is thus an important stage. Such validation has to show how well the elements of interest are detected and how robust each algorithm is to variations of signals, e.g. the ongoing background (BKG) activity or the numbers of events. Most HFO detectors were validated using real data [4, 5, 7, 9], based on visual marking, using marking by an expert as ground truth. The validation process typically consists in three steps [4, 5, 7, 9]: firstly, expert reviewers mark events of interest, i.e. ripple (R, 80-250Hz) and fast-ripple (FR, 250-500Hz), on a recording; then the detector is run over the same data and finally the detections are compared to the reviewers’ marking and its performance is measured.

This strategy however suffers from several issues. The main drawback is that there is no gold standard: is an HFO marked by the detector but not by the reviewers necessarily wrong? It could be that the detector extracts a feature that is not evident for the reviewers. Without gold standard, this situation will always be considered as an erroneous detection. Furthermore, the performance highly depends on the content of the tested signal. On the one hand, the performance is measured as a global agreement over two classes (R and FR) between the detections and the reviewers’ marking. Since there are more Rs than FRs [11] and that Rs are probably easier to detect than FRs due to their higher amplitude and longer duration [4] and the 1/f spectrum, the performance measure does not reflect the actual efficiency of the detector on the R and FR class separately. All the FRs could be missed but the performance of the detector could still be high because of this imbalance. Since the FRs are a putative better marker of epileptic tissues [1, 12, 13] this may be a critical issue. On the other hand, robustness cannot be studied in real data since the content is fixed. For instance, aforementioned detectors rely on thresholds which are calculated using measures such as standard deviation or percentile. These measures depend on the distribution of the data and therefore on the content of the recordings (number of spikes and number of HFOs). Moreover, if detectors were tested on recordings with high amplitude HFOs, the results cannot be extrapolated to cases where the amplitude of the HFOs is closer to the amplitude of the BKG.

Realistic simulations may be a solution to these drawbacks. The balance between the classes can be controlled and the gold standard is obviously available. We propose a method that aims at building a dictionary of representative extracted events (Spike, R, FR) from different brain areas and integrating them in a controlled manner in a simulated BKG from the same area to determine the performance and robustness of the detectors. In a second step, we show how to use the results of this benchmark to diagnose currently-available detectors, in term of assets and weaknesses.

## Methods

### Clinical database

Simulated data were created by using recordings of non-REM slow wave sleep of drug resistant epileptic patients undergoing pre-surgical examination with stereoelectroencephalography (SEEG) using macro-electrodes of diameter 0.8mm. The same type of electrodes was used in all patients. The frequency sampling was 2048Hz with an anti-aliasing filter set at one third of the sampling frequency (688Hz). We only selected contacts which were inside the epileptogenic zone defined by expert neurophysiologists (FP and FB). Selected channels had to exhibit interictal spikes, ripples and fast-ripples on bipolar montage. Explored brain areas included the mesio-temporal, lateral temporal, frontomesial and frontolateral regions. We did not simulate posterior regions because they are under-explored and little is known about HFOs in these areas.

### Simulation

#### Extraction of events

**Fig 1:**
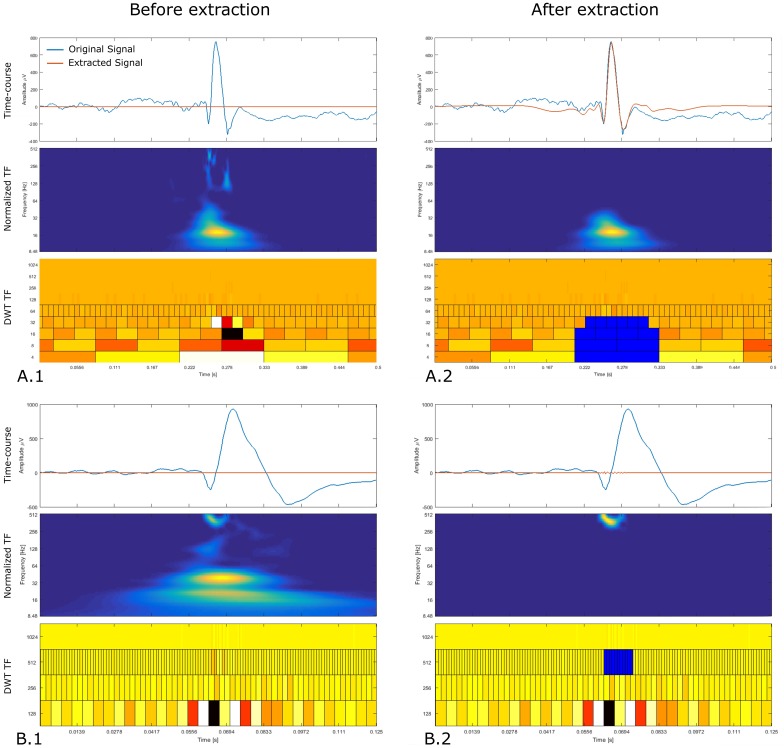
Event extraction illustration. Each panel is composed, from top to bottom, of the original signal (blue) with the synthesize signal (orange), the normalized continuous TF image and the discrete TF image. Panels A and B display the process of extracting an epileptic spike and a fast-ripple riding a spike, respectively, at the beginning (.1) and the end (.2) of the process. We also provide an animated version of these two panels here: http://meg.univ-amu.fr/wiki/AnyWave:Plugin_Simul. User selects the coefficients of interest in the discrete TF image by left-clicking the appropriate tile. Once a tile is selected, it turns blue and the inverse transform is applied to the corresponding coefficients in order to reconstruct the signal. At the beginning of the process, the continuous TF represents the TF of the original data and during the process it shows the TF of the extracted signal.

To build the dictionary of representative events, we only used visual marking and no automatic detection to avoid favoring one detector. We visually marked the events of interest on a bipolar montage according to [14]. We extracted them from the signal using the following method. We developed a Graphical User Interface (GUI) which displays in three different panels simultaneously the raw signal with the extracted signal, the normalized time-frequency (TF) obtained using Continuous Wavelet Transform (CWT) and, finally the TF relative to the discrete wavelet transform (DWT) (Wavelet Toolbox of MATLAB). By selecting the relevant coefficients of the DWT TF, one can reconstruct parts of the signal and thus extract elements without the surrounding BKG. If elements were superimposed, i.e. a HFOs was superimposed on a spike or a FR on a R, we reconstructed the R, FR and spike separately by selecting the appropriate DWT coefficients. We skipped the elements which were overlapping both in time and frequency [15]. Two examples of extracted events are given in Fig 1. To avoid introducing unwanted oscillations, the user ensured both in normalized TF and in the original and synthesized band-pass filtered signals the correct extraction of the elements.

This paradigm allows us to control the Signal to Noise Ratio (SNR) of each event separately according to their own frequency band. This also guaranty the stability of the BKG of the simulation which would not have been the case if we had cut and added real elements which would have ineluctably carried a part of human BKG.

HFOs could have been simulated by tapering sine waves of specific frequencies as it was done in [16, 17] but we wished to keep the original shape of the HFOs as it was recorded to build a dictionary relative to the structure. This also allows us to use frequency and amplitude modulations found in real HFOs which makes the simulation more realistic. For the same reasons, we used this technique to extract spikes.

#### Background simulation

**Fig 2:**
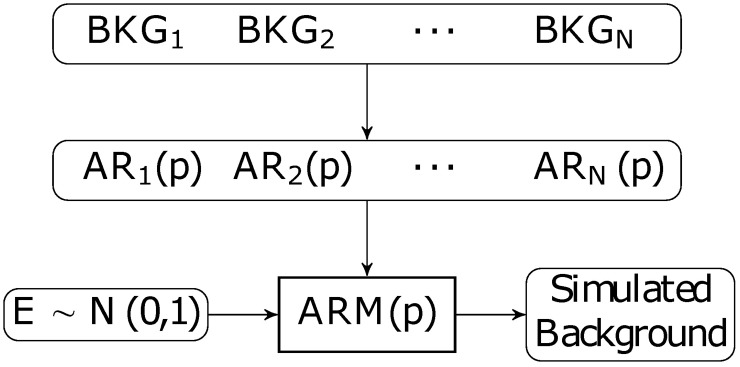
Background simulation pipeline. BKG segments are taken from the given BKG sections, the AR coefficients are estimated for each BKG section and averaged. The simulated BKG are obtained by filtering a Gaussian white noise with the average AR coefficients.

The ongoing background activity (BKG) of the areas where the HFOs and spikes were extracted were simulated. This step is useful to verify the robustness of the algorithm to variability in BKG activity across brain structures. We marked several baseline sections of the recording, i.e. containing only BKG activity. Since errors may occur, reviewers were asked to mark several pieces of baseline of several second for each channel of interest. The coefficients of an autoregressive (AR) model were estimated for each section (e.g. Matlab’s LPC function) and averaged over the pieces of the same channel to have an estimate of the BKG for each channel. To generate the simulated BKG activity, we filtered a Gaussian white noise with the averaged AR coefficients. Every BKG is generated using a new realization of the Gaussian white noise. Fig 2 represents a schematic view of the BKG simulation.

#### Simulation generator

Once the events and BKGs of the different areas are extracted, they can be added together in a controlled manner by the following procedure. This generator aims at simulating seven classes of events corresponding to: 1. Spike (Spk), 2. Spk co-occurring with a R (Spk-R), 3. Spk co-occurring with a FR (Spk-FR), 4. Spk co-occurring with a R and a FR (Spk-R-FR), 5. R, 6. FR and, 7. R co-occurring with a FR (R-FR).

**Fig 3:**
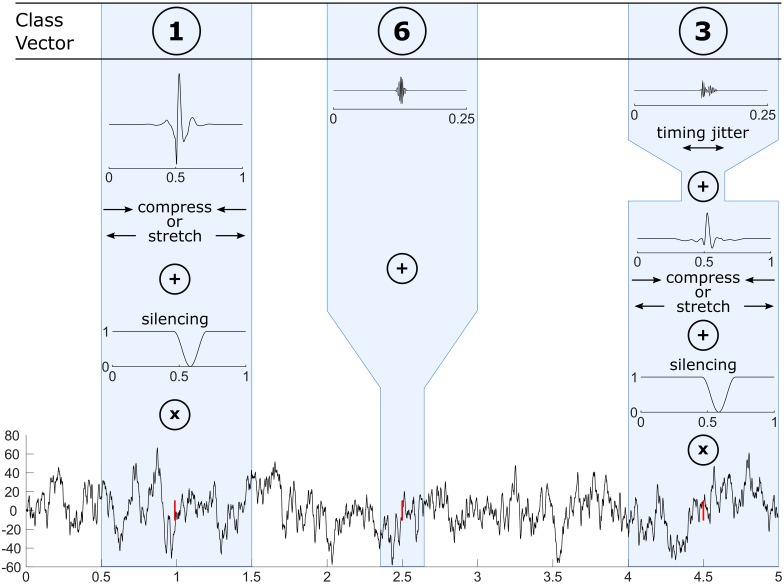
Event insertion pipeline. The class vector contains a set of randomly drawn numbers corresponding to the event classes for each timing (red line). At 1, 2.5 and 4.5s, events of the first (Spk), third (FR) and sixth (Spk-FR) classes are added to the BKG. Each Spk is stretched or compressed and scaled to fit a random SNR (ranging from 0-15dB). The BKG is multiplied by a notch to reproduce post-spike silencing. HFOs are scaled to fit the chosen SNR and have a timing jitter when riding a Spk.

**Fig 4:**
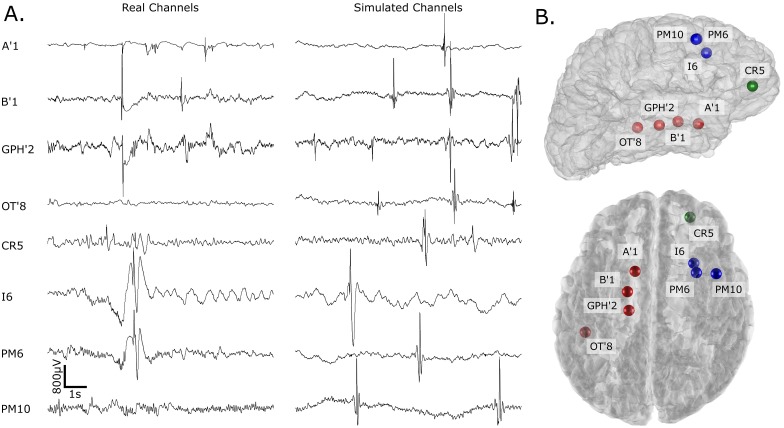
Example of real data and their corresponding simulations with their 3D localization. Each color corresponds to a different patient.

**Fig 5:**
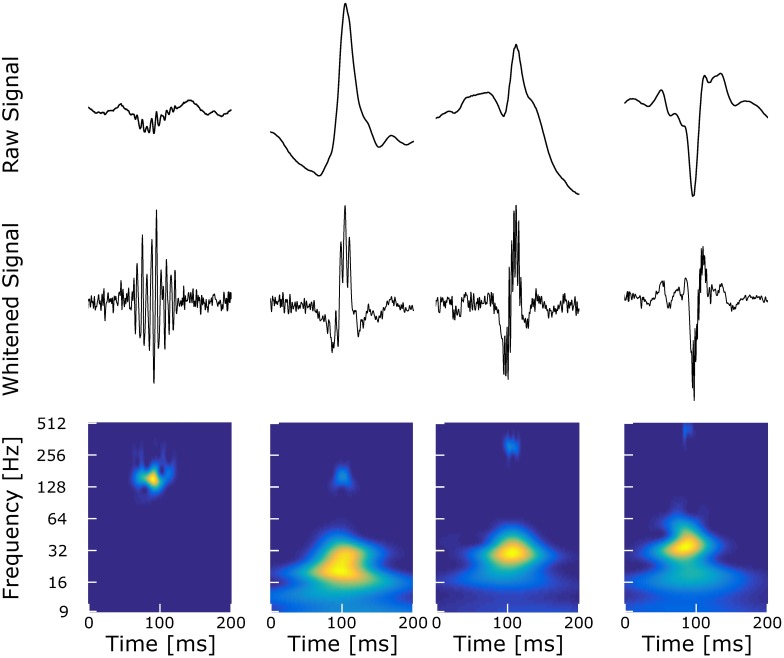
Example of simulated HFOs at 15dB (R, Spk-R, Spk-FR, Spk-FR). Each column is composed, from top to bottom, of the raw signal, the whitened reconstructed signal and Z_*H*_0__ normalized TF image [17]. Note that in the raw signal the HFOs above are hidden in the slope of the spikes. There is however no doubt about their existence since there were manually added. Also observe that the whitened signal unveils these HFOs while preserving the shape of the spikes.

Using the same GUI, the user sets the rates of each class, the values of SNRs and the number of realization per simulated channel and per SNR. The event class are randomly drawn according to the input rates for each timing. Finally, the events are selected randomly in the dictionary of the channel of the patient according to the selected class. Spks and HFOs are added using different processes. First of all, the SNR of the HFOs is fixed for each realization of the BKG whereas the SNR of the Spk varies randomly. Each inserted Spk is first stretched or compressed by a pseudo-random factor k, scaled to fit the randomly selected SNR and inserted in the BKG. By doing so, we can create spikes which exhibit a high-frequency component and reproduce the overlap in time and frequency of HFOs superimposed on a spike which we could not extract properly as explained in section Simulation. Moreover we simulated the post-spike silencing [18] by reducing the amplitude of the BKG during the Spk. This generates non-stationarity in the BKG and challenges the estimation of features used for the threshold. Each inserted HFO is first scaled to fit the fixed selected SNR in its relative frequency band and added to the BKG. Globally the timing of the events follows a random Poisson process. Locally, we added a timing jitter to the HFO when superimposed to HFO or spike. An example of the insertion of the elements is given in Fig 3. Fig 4A illustrates real channels facing their simulated versions, Fig 4B displays the location of the simulated channels in a 3D brain mesh and Fig 5 shows examples of simulated HFOs. We generated a set of simulations with a rate of 3/min for each event class. It contains 30 realizations of each channel for SNRs set to 0-5-10-15dB

### Performance evaluation

Once the simulations have been generated, detectors can be run. We defined a small time window of 100ms centered on each inserted HFO as the confidence interval (CI). CIs containing detections were considered as True Positives (TP), those without detections were defined as False Negatives (FN) and, detections falling outside CIs were labeled as False Positives (FP). We use the precision (Prec) and sensitivity (Sens) criteria as well as the F-measure which combines Prec and Sens to characterize the performance over the SNRs. We avoid using the specificity because of the issue of the imbalance in the distribution of positive and negative instances [19] and because the True Negative are not clearly defined. Sens and Prec are defined as follows

$$\begin{array}{c} Sens=TPR=TP/P=TP/(TP+FN), \end{array}$$ (1)

$$\begin{array}{c} Prec=PPV=TP/(TP+FP). \end{array}$$ (2)

We also computed the sensitivity per class to determine whether the quality of detection was class dependent.

### HFO detectors

Five automatic detectors were tested; four of them come from the RIPPLELAB Toolbox [16] which we integrated in our open source software AnyWave [20]. The fifth detector is our detector called Delphos (Detector of ElectroPhysiological Oscillations and Spikes) described in [17]. The next paragraph will briefly summarize the different detection methods.

The Short Time Energy detector (STE) [4] is based on the moving average of the root mean square amplitude of the filtered signal. The segments above five times the standard deviation (SD) plus the mean energy lasting more than 6ms are considered as putative HFOs. These events are kept if containing more than 6 peaks greater than 3 times the SD above the mean value of the rectified band-pass signal.

The Short Line Length detector (SLL) [5] calculates the line length energy of a sliding window applied to a first-order backward differencing and band-pass filtered signal. A detection is retained if its amplitude is greater than the 97.5th percentile of the empirical cumulative distribution function and longer than 12ms.

The Hilbert detector (HIL) [6] computes the envelope of the filtered signal using the Hilbert Transform. The local maxima exceeding 5 SD of the envelope of the whole signal with a minimal time length of 10ms are labeled as HFOs.

The MNI detector (MNI) [7] is a two-stage algorithm. Firstly, baseline segments are detected using the wavelet entropy of the auto-correlation function of the band-pass filtered signal. If there is enough baseline segments, the detection procedure is similar to the STE detector except that the threshold is set to the 99.9999 percentile of the empirical cumulative distribution function of the baseline segments. Otherwise, the STE detector is run with a threshold optimized by iteratively removing previously detected HFOs. We modified the algorithm of RIPPLELAB concerning this detector to be closer to the original one described in [21].

All the parameters of the detectors correspond to the default setting of RIPPLELAB which corresponds to the one used in the respective articles. The signals were bandpass filtered between 80-500Hz for all detectors as in the original publications, except for Delphos which does not rely on filtered signals.

Delphos detects oscillations and spikes in the Z_*H*_0__ TF [17] representation by analyzing the time width and frequency spread of peaks above a threshold. The values in the Z_*H*_0__ TF correspond to an normalized energy. We set the threshold to 30 in the Z_*H*_0__ TF. Detections are classified as oscillation, if their frequency spread is similar to the one of the wavelet and their time width is greater than the one of a Dirac impulse, or as spike if their frequency spread is greater than the one of the wavelet and their time width is similar to the one of a Dirac impulse.

## Results and discussion

**Fig 6:**
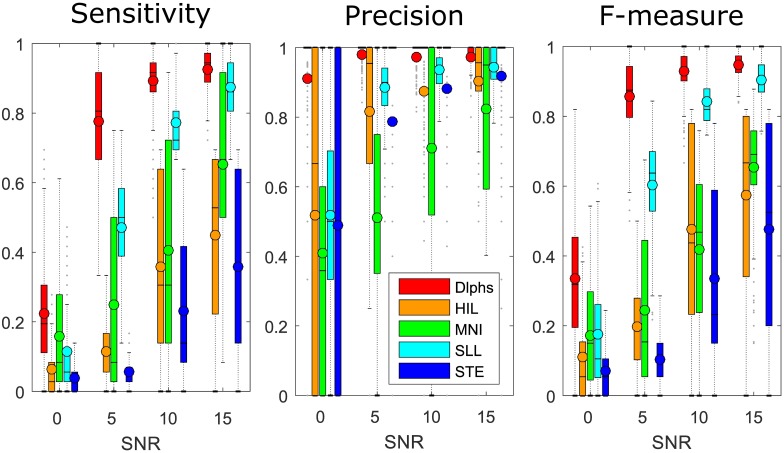
Boxplots of the sensitivity, precision and F-measure of the detectors ran over this benchmark for different SNRs. The circles correspond to the means and the dots represents the outliers. The sensitivity of all detectors increases with the SNR. Interestingly, the precision also increases with the SNR except for Delphos whose precision stays at 1. The F-measure combines the sensitivity and precision.

**Fig 7:**
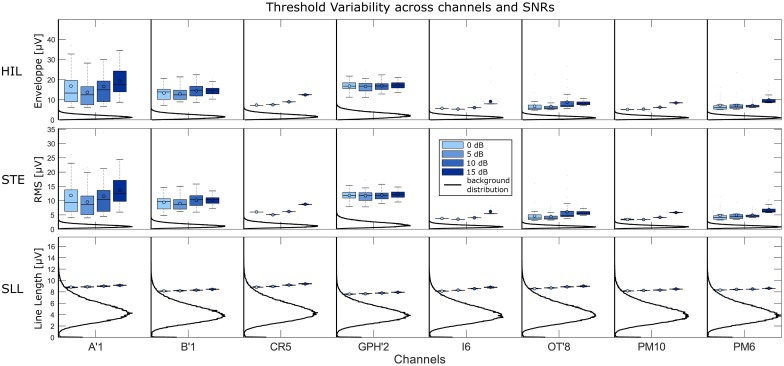
Thresholds determined by the detectors compared to the background. Boxplots of thresholds for the different metric (RMS, SLL and hilbert enveloppe) used by the one-stage detectors (STE, SLL and HIL) compared to the histogram of the metric applied to the simulated background.

Fig 4 illustrates the realism of our simulation. Note the similarity between the simulated BKG and its original form and its variability. Moreover the shape of the spikes and the HFOs are more complex and diverse than previous simulations [16, 17, 22], which is more realistic. Finally the structure of the simulation, i.e. the statistical similarity of the BKG corresponding to each channel, the control of the SNR and the control of the rates of every classes enables us to test the robustness of the detection. According to the design of the simulation, one can make some assumptions on the results. First of all, the sensitivity should increase with increasing SNR. For 0dB, the amplitude of the HFOs is similar to that of the BKG; consequently the sensitivity should be close to zero. Moreover, the variability of the precision should be greater than for other SNRs because of the low sensitivity. For 15dB however, the amplitude of the HFOs is a lot higher than the one of the BKG; consequently the sensitivity should approach one. This is consistent with our results (Fig 6 and S1 Fig), where the sensitivity of all detectors increases with the SNR. However, some detectors (MNI, HIL, STE) do not reach 1 in sensitivity. This observation is partly explained by the fluctuation of the threshold of the STE, HIL, SLL detectors (Fig 7).

### Robust estimation of the background activity

In Fig 7, we see that all thresholds are increasing with the SNR, especially for the HIL and STE detectors. This is consistent with the fact that they both rely on SD to calculate their threshold which leads to a biased estimation of the BKG boundary since the events weight more in the histogram for higher SNRs. The SLL nevertheless uses a percentile threshold which is more robust to outliers and thus more stable but still slightly increasing with the SNR. Note that this can be studied only because the simulated BKGs are statistically identical for each channel since it was generated using the same AR coefficients and white noise with the same properties. In other words, different realizations of a BKG from the same simulated channel yield the same histogram. Consequently the increase of the thresholds is only due to changes in amplitudes of the HFOs. Moreover, the STE and HIL thresholds are high compared to the tail of the BKG distributions; the threshold could be lowered to increase the sensitivity without losing in precision. Interestingly, the variability of the HIL and STE thresholds is the largest for all SNRs in A’1, B’1 and GPH’2, which are the channels recording from the amygdala, hippocampus cephalis and hippocampus caudalis, respectively. In these channels, we found the sharpest spikes, which created large outliers in the distributions. This shows again how the SD can be biased by outliers and how the percentile method of the SLL is more stable. Nevertheless, the percentile will always give a value in the range of the data; this means that if there is only BKG activity, the threshold will still select portion of the BKG as putative HFOs. These issues highlight the need of estimating the threshold based on the histogram of the BKG—the null hypothesis H_0_—and not on the histogram of the mixture (BKG plus events; H_0+_H_1_). This is what the MNI detector and Delphos aim at solving by either finding baseline/BKG sections or estimating robustly the BKG activity, respectively.

**Fig 8:**
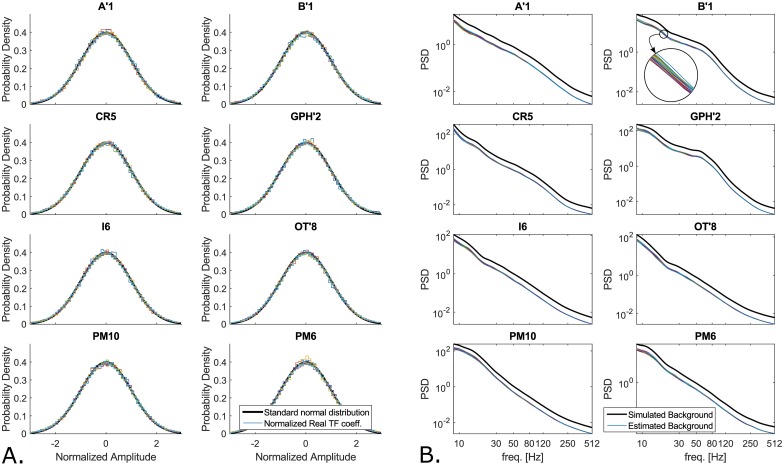
Estimation of background activity and spectra of different backgrounds calculated by Delphos on the simulated channels. A. shows in each panel the probability density function of a standard normal distribution (*μ* = 0 and *σ* = 1) in solid black line and the histogram of the normalized real coefficients of the TF at different frequencies calculated for one realization at 15dB of each simulated channels. The quality of the estimation of the BKG activity can be assessed by comparing the closeness of these histograms to a standard Normal distribution. B. represents the estimated BKG spectra (color coded) retrieved by Delphos from all the realizations of the simulated channels and the actual spectra of the simulated BKG of these channels (black line). We added an offset to the simulated BKG spectra to avoid overlapping of the estimated and the actual spectra. In B’1 panel, we added an insert showing the small variability of the method.

While Delphos uses the Z_*H*_0__ TF normalization to estimate the BKG activity, the MNI detector finds baseline/BKG segments based on wavelet entropy (WE). In theory, both techniques are similar since the threshold is calculated on either a robust estimate of the BKG histogram derived from the total histogram (Delphos) or on the histogram of the detected BKG segments (MNI detector). Delphos estimates the BKG activity at each frequency by fitting a Gaussian distribution on the histogram of the real coefficients of the wavelet transform within the Tukey’s range (for more details please refer to [17]). One can estimate the power of the BKG by squaring the obtained SD and also normalize the TF plan by z-scoring each line with the same SD. In Fig 8, we can see that each histogram follows a standard Normal distribution—this shows that the estimation stage was effective—and that the spectra of the estimated BKGs computed on the simulated channels thoroughly follow the spectrum of the original BKG signal independently of the whole frequency content. The threshold was set to 30 which correspond to $thr=\sqrt{30}\sigma =\sqrt{30}$ and is thus stable since the estimation is robust. On the contrary, the WE calculated on the auto-correlation function of the filtered signal depends on the power spectrum density (PSD). In Eq (14) of Appendix A, we show that applying the wavelet transform to the auto-correlation function is equivalent to applying it to the PSD of the filtered signal in the frequency domain. In other words, the values of the WE changes with the power of the spectrum whether there is rhythmicity, i.e. oscillations, or stronger broad-band activity.

**Fig 9:**
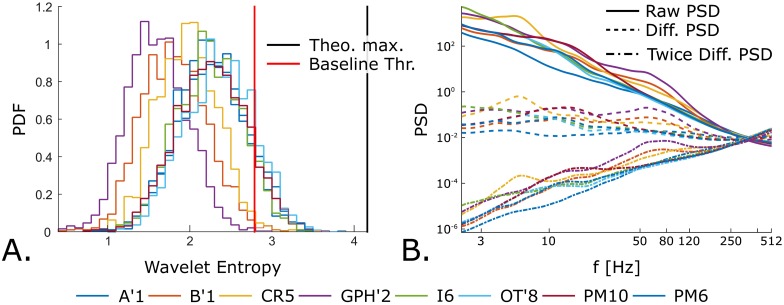
Wavelet Entropy (WE) of the simulated background of each channel and their differentiated power spectrum density. A. histograms of the minimum WE calculated on the autocorrelation function of the filtered simulated BKG (without inserted events) of each simulated channels. The theoretical maximum and the threshold value are represented in black and red lines respectively. B. Power spectrum densities (PSD) of the simulated BKG activity and the effect of differencing. Colors refer to the same channels in A. and B. Note that the histograms which have the fewest number of sections above the threshold values (A.) correspond to B’1(orange), CR5(yellow) and GPH’2(purple) which exhibit the highest power in the 80-500Hz band (B.). Also observe that (i) the differentiated spectra (B., dashed line) is flatter than the raw spectra but not completely flat and that (ii) the 2nd-order differentiated spectra (B., dashed and pointed line) inverse the slope and over-express the high-frequencies.

The histogram of the minimum WE of all the sections (Fig 9) demonstrates a shift for BKGs with higher power in the 80-500Hz band. This shift is interpreted as channels with semi-continuous high-frequency (SCHF) activities and the MNI detector switches into the no baseline mode. Interestingly, this shift occurs in the B’1, GPH’2 and CR5 simulated channels which correspond to the synthesized hippocampus cephalis, hippocampus caudalis, and the cingulate region. These regions are prone to generating SCHF activities [23, 24] and this illustrates again the realism of our simulation. The idea underlying the concept of SCHF activities is that, in those channels, it is harder to visually identify HFOs due to this active BKG. Therefore, to increase the sensitivity, the threshold has to be optimized to be closer to the BKG. Our simulation reproduces this context for the three aforementioned channels at 0dB. In this context, the MNI detector exhibit the best performance in sensitivity compared to other detectors but have the worst precision (S1 Fig). The main issue is that the MNI remains in this “no baseline mode” even when the HFOs are distinguishable from the BKG (SNR> 0dB). It still performs well in term of sensitivity, even though it is caught up by Delphos and the SLL detector, but has the worst precision among the detectors. This is of clinical importance since R occurring in flat BKG seems to correlate more with seizure freedom than R occurring in oscillatory BKG [25]. Despite this low threshold, Delphos and the SLL detectors manage to have an equal or better sensitivity generally but especially in those channels compared to the MNI detector. This introduces the second result of this benchmark; namely that detectors do not have the same sensitivity across the classes.

### Accounting for the power spectrum

**Fig 10:**
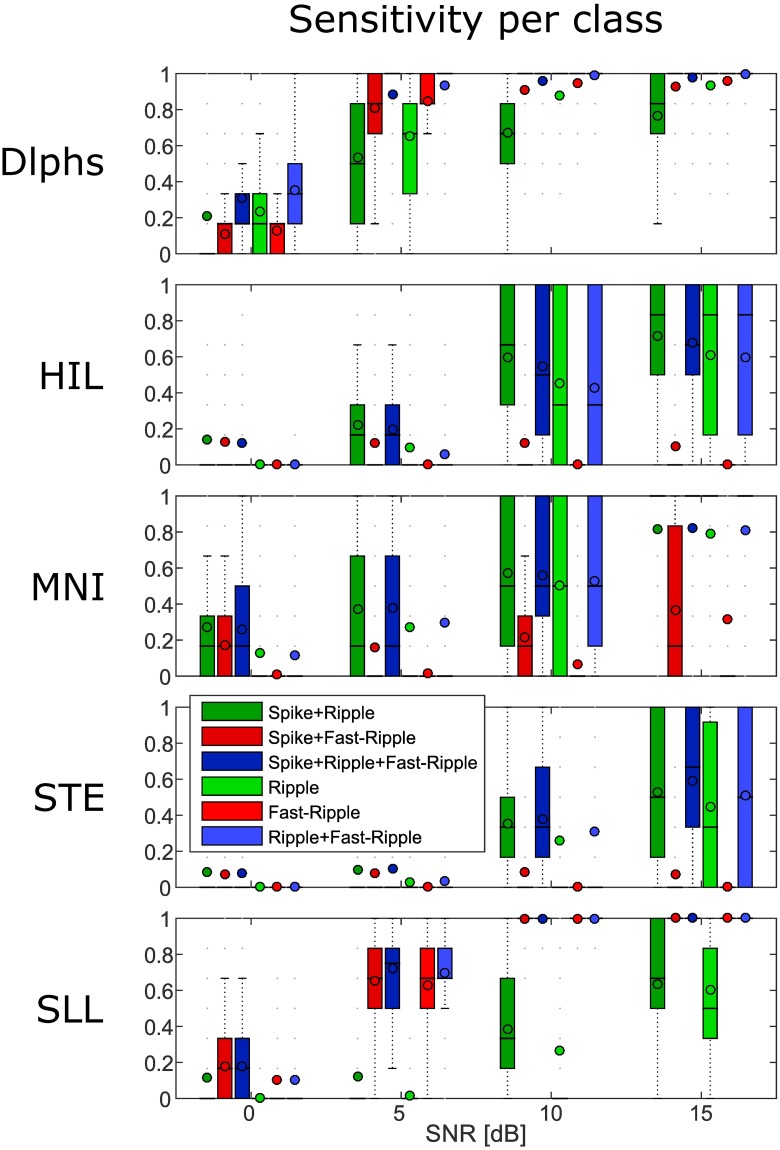
Boxplots of the sensitivity per class for each detector. Globally, the sensitivity per class increases with the SNR. All detectors have issues detecting ripple riding spike. The HIL,MNI and STE detectors are more sensitive to the ripple classes thant to the fast-ripple classes. The SLL has a better sensitivity in the FR band than in the R band. Delphos has a comparable sensitivity per class at each SNR.

The MNI, HIL and STE detectors have a better sensitivity to the classes containing R (Spk-R, Spk-R-FR, R, R-FR) than to classes with FR only (Spk-FR and FR) (the second to the fourth panel of Fig 10). The SLL detector is the opposite; it has high sensitivity to FR classes (Spk-FR, Spk-R-FR, FR, R-FR) but low sensitivity to classes with R only (Spk-R, R) (last panel of Fig 10). Delphos seems to have either an equal or better sensitivity per class except for the Spk+R which is somehow lower than the other classes (first panel of Fig 10). The first statement is explained by the 1/f structure of the physiological data. In our simulation, the SNR of the HFOs is constant for each realization and set according to the level of the BKG in the frequency band of the added HFOs, i.e. in the R or FR band. The amplitude of a R is therefore higher than that of a FR for the same SNR; the faster the oscillation, the smaller its amplitude [26, 27]. Determining a threshold in the 80-500Hz band is thus suboptimal. Lowering the threshold to increase the sensitivity in the FR band would increase the number of false detections due to the higher BKG in the R band. A solution would be to run the detector on the R and FR band separately as was done in [13, 28]. The slope of the spectrum still remains in each band but it undoubtedly improves the results for a longer computing time.

A solution is to whiten the spectrum, i.e. to flatten the spectrum in order to balance the power across frequencies as done by the SLL detector and by Delphos. However the latter performs better for each class compared to the SLL. This is due to the quality of the whitening stage. Delphos uses the Z_*H*_0__ TF whitening as discussed above in section Robust estimation of the background activity. In [17], we showed that it was as efficient as normalizing using a baseline. The main advantage of the Z_*H*_0__ normalization is that it adapts to the shape of the BKG (Fig 8) whereas the pre-whitening stage of the SLL is fixed. Moreover, we showed in [17] that the pre-whitening by differencing was not optimal but would not lead to over-express the higher frequencies. In theory the SLL detector should detect the R as well as the FR since its threshold is robust and a pre-whitening stage is applied, In fact, in Eq 6 of Appendix A, we derive that the line length metric used to detect the HFOs is equivalent to a second differencing stage, which over-expresses the higher frequencies (Fig 9B). This explains the very high sensitivity in the FR band and the poor sensitivity in the R band. Furthermore, the differencing method is dependent on the sampling frequency (Eq 8, Appendix A), i.e. the pre-whitening stage will not have the same effect if applied to data recorded at 1024Hz or 2048Hz. This is an important aspect to consider when discussing clinical results.

Globally, every detector has issues with detecting R superimposed on Spk when they are overlapping both in time and frequency. The main difficulty for detectors using time-series is to separate actual Rs riding on Spks from filter ringing [22]. In [29] a solution was proposed based on feature extraction and unsupervised clustering but this method is not yet implemented in current detectors. In this latter article, the authors also highlight a potential problem of TF analyzes. Because of the structure of the analysis, the Spk could hide the HFOs. In fact, Delphos which detects HFOs in TF plane suffers from this masking but still outperform the others. This is probably due to the fact that masking only occurs for a very specific value of the phase and thus generate a single peak in the TF image instead of two separate. A possible future step would be to subtract the spike waveforms before running the detectors [30, 31].

Moreover, even though Delphos seems to give good results, it has some drawbacks. For instance, it misses HFOs with strong frequency modulation (chirp). This type of HFOs create a “comma-like blob”—similar to the FR shown in Fig 1—which makes the calculation of the time width difficult. A solution may be to calculate the width using the ridge to have a better estimate. This would probably increase the computing time greatly but the significant increase in sensitivity has yet to be proven. One could also add features to describe the “blob” such as its surface or volume but it would be difficult to compare it to a theoretical model.

### Clear definition of HFOs

In previous paragraphs, we focused on the sensitivity (number of TP); we will now concentrate on the precision (impact of the FP on the detection). One key prerequisite of a detector is that its precision should be constant across the SNRs, i.e. it should not have more FP at lower SNR than and at higher SNR and inversely. This involves two factors, a robust threshold—we discussed that in section Robust estimation of the background activity but also a strict definition of HFOs [32].

Most detectors defined them as a sustained increase in energy in the HFO band (SLL, HIL, MNI, STE). The STE detector adds the criterion of the number of cycle. It was however shown in [22] that filtering artefact could produce sustained oscillations of high amplitude. The aforementioned criteria lack in distinguishing these false ripple from real ones and thus decrease their precision (Fig 6 and S1 Fig). Also the duration threshold is an inadequate criterion. For the STE, it was fixed at 6ms which corresponds to the duration of 3 periods of a 500Hz oscillations. A 2-cycle oscillation at 250Hz has a longer duration than 6ms but do not fall under the definition of a HFO. To be efficient, this duration threshold should be calculated and applied after determining the frequency of the oscillation in order to fit the 3 or 4 cycles criterion. Naturally, one can think of the TF analysis for quantifying this duration at a specific frequency. Delphos uses this technique to validate the cycle criteria. Moreover it takes advantage of a practical property of the log-scale wavelet TF image, namely that oscillations have the same frequency width whatever their frequency. The definition of an HFO in the TF image is an increase in the TF representation which is wide enough in time but has a limited spread in frequency. This definition enables Delphos to have a high and stable precision for all SNR. Two-stage detectors such as [9] also use a TF analysis in the second stage to increase the precision on previously detected HFOs. This however only increases the precision but does not solve the issues, previously discussed in sections Robust estimation of the background activity and Accounting for the power spectrum, concerning the sensitivity of the first stage detector.

### Clinical implication

To push forward HFOs into a clinical context, one should know the exact features and limitations of the detectors. Imagine a study which aims at mapping the rates of the Rs and FRs in various brain structures using a detector with a better sensitivity in the R band than in the FR band. Quantifying its performance using only visual marking would not have revealed this bias and thus the clinical conclusion could have been that structure *A* never exhibits FRs even thought it does. The lack of characterization could lead to contradictory results. Team *1* uses detector *A* while Team *2* uses detector *B* which have a better sensitivity in FR than in R. After running their detector on a large cohort of patients, Team *1* would conclude that Rs are betters marker of the epileptic zone than FRs whereas Team *2* would say the opposite. Both are right if you consider only statistical significance; but both are wrong because they did not take into account the detector biases.

Höller and collaborators [33] proposed to validate automated detectors against outcome instead of visual detection. In a scientific perspective, it seems to be an ambiguous idea. Assume that there is a HFO detector that succeeds in predicting the outcome. We would know which part of the brain to resect but we would not know why. We would have found the perfect outcome detector but not the perfect HFO detector. This detector could have detected “false” HFO [22] resulting from very sharp spikes or oscillations contained in the semi-continuous high-frequency activity [24]. All these features could indeed be a manifestation of epileptogenesis but in the perspective of treating the patients with less invasive method, we would not know what to target… However, combining sensitive and precise detectors of all types of epileptic features to generate a meta-detector to predict the outcome could be an interesting goal. This way we could understand how the different features are related to epileptogenicity.

Concerning the separation between physiological and pathological HFOs, we did not address this delicate question because in our simulation we only used HFOs from epileptogenic regions which are likely to be pathological. We however believe that all HFOs have to be detected and, only in a second stage, classify as physiological or pathological using extra features, e.g. amplitude, number of cycles. The main issue concerning this classification is that no clear feature has been defined yet. Therefore this benchmark only assesses the performance of the detectors on their capability to detect HFOs, whether pathological or physiological, and does not presume to bind the performance of a detector with the surgical outcome of a patient.

## Conclusion

The present paper describes a framework of simulations which shows the possibility of reproducing SEEG signal realistically in a controlled manner. By extracting elements of interest from their original BKG using DWT and by simulating their surrounding BKG using AR coefficients, we were able to reproduce signals from different brain areas while controlling the SNR of each added elements of interest separately. This control enabled us to study the performance and robustness of the detectors to several variations, i.e BKG variability, modification in amplitude and type of the elements.

Our simulation study has highlighted weaknesses and assets of several detectors that were never mentioned in previous studies as far as we know. They have pointed out:

1. the instability of the threshold to distinguish HFOs from background due to the unstable approach to estimate the background activity (section Robust estimation of the background activity).
2. the underestimated impact of the 1/f spectrum (section Accounting for the power spectrum) which causes a difference in the class sensitivity,
3. the fuzzy definition of HFOs which leads to a decrease in precision (section Clear definition of HFOs).

To solve these issues we propose (1) to estimate the BKG activity by either detecting sections of baseline/BKG or by extracting its characteristics from the whole signal, (2) to take into account the shape of the spectrum by, for example, whitening the signal and finally (3) to use a strict definition of HFOs to avoid detecting events which look alike but originate from different entities.

We encouraged other groups to take part in the construction of a wider simulation set by either providing data of other brain areas and modalities or by generating their own simulation. This benchmark will be uploaded in open-access on the AnyWave (http://meg.univ-amu.fr/wiki/AnyWave:Plugin_Simul) website as well as the simulated SEEG data and the detection markers of the detectors. We believe that, by building such a broad and cross-modality simulation set, we will answer some unsolved questions concerning HFOs.

**Appendix A** Let *x*, $\tilde{x}$ and Γ be the signal, the Fourier transform of *x* and the auto-correlation function of *x*, respectively. The power spectrum density (PSD) of *x* is defined as

$$\begin{array}{c} \Sigma ={\left|\tilde{x}\right|}^{2}. \end{array}$$ (3)

The first stage of the SLL detector is a first-order backward differencing filter. We showed in [17] that this process, which corresponds to a pre-whitening stage aiming at flattening the 1/f spectrum, does not completely flatten the spectrum but does not over-express the high frequency. In fact, the algorithm of the SLL detector can be rewritten to highlight the fact that, indeed, the short line length metric correspond to a second differencing filter. Let *x*, *x*_1_, *x*_2_ and *n* be the original signal, the differentiated signal, the second order differentiated signal and the sample.

$$\begin{array}{c} \begin{array}{c} x_{1}\left[n\right]=x[n]-x[n-1],\\ x_{2}\left[n\right]=(x[n]-x[n-1])-(x[n-1]-x[n-2]),\\ x_{2}\left[n\right]=x_{1}\left[n\right]-x_{1}\left[n-1\right]. \end{array} \end{array}$$ (4)

The SLL energy expression for a time window *W* is

$$\begin{array}{c} E_{SLL}\left[n\right]=\sum_{k=n-W+2}^{n}\left|x_{1}\left[k\right]-x_{1}\left[k-1\right]\right|, \end{array}$$ (5)

and using Eq 4

$$\begin{array}{c} E_{SLL}\left[n\right]=\sum_{k=n-W+2}^{n}\left|x_{2}\left[k\right]\right|. \end{array}$$ (6)

We therefore derive that a first order differencing stage followed by a SLL energy calculation is proportional to a rectified moving average of a second order differencing signal. By applying a second order differencing, higher frequencies are more enhanced and the power spectrum almost inverses its slope. This explains the very high sensitivity in the fast-ripple band and poor sensitivity in the ripple band. Furthermore, the differencing method is dependent on the sampling frequency which appears clearly when we study the discrete Fourier transform

$$\begin{array}{c} \text{DTFT}\left\{x_{2}\left[\;.\;\right]\right\}={\left(1-e^{-j\omega }\right)}^{2}\tilde{x}\left(e^{j\omega }\right), \end{array}$$ (7)

where *ω* = 2*πf*/*f*_*s*_ is the normalized frequency and *f*_*s*_ the sampling frequency. Its PSD thus is

$$\begin{array}{c} \begin{array}{cc} {\left|\text{DTFT}\left\{x_{2}\left[\;.\;\right]\right\}\right|}^{2} & ={\left|1-e^{-j\omega }\right|}^{4}\Sigma \left(\omega \right)\\ & =2\left(3+\cos\left(2\omega \right)-4\cos\left(\omega \right)\right)\Sigma \left(\omega \right) \end{array} \end{array}$$ (8)

The baseline detector is based on wavelet entropy (WE). The Continuous Wavelet Transform (CWT) of a signal *y* is defined as

$$\begin{array}{c} T_{y}\left(b,a\right)=\frac{1}{\sqrt{a}}\int_{-\infty }^{+\infty }y\left(t\right)\bar{\psi \left(\frac{t-b}{a}\right)}dt, \end{array}$$ (9)

with *ψ* the wavelet function, *a* the scaling factor, and *b* the shifting factor. The wavelet entropy is defined as

$$\begin{array}{c} S\left(b\right)=-\sum_{k\in A}P\left(k,b\right)\log_{10}\left(P\left(k,b\right)\right), \end{array}$$ (10)

where *A* is the set of scales and *P* is the normalized wavelet power which is similar to a probability and corresponds to

$$\begin{array}{c} P\left(a,b\right)=\frac{{\left|T_{y}\left(a,b\right)\right|}^{2}}{\sum_{k\in A}{\left|T_{y}\left(k,b\right)\right|}^{2}}, \end{array}$$ (11)

Let us show how the WE is dependent on the PSD and thus the profile of the 1/f spectrum. Applying Eq 9 to the auto-correlation Γ as it is done in the MNI detector, we have

$$\begin{array}{c} T_{\Gamma }\left(b,a\right)=\frac{1}{\sqrt{a}}\int_{-\infty }^{+\infty }\Gamma \left(t\right)\bar{\psi \left(\frac{t-b}{a}\right)}dt, \end{array}$$ (12)

then using the Parseval theorem [34] it yields

$$\begin{array}{c} T_{\Gamma }\left(b,a\right)=\frac{1}{2\pi }\sqrt{a}\int_{-\infty }^{+\infty }\tilde{\Gamma }\left(\xi \right)\bar{\tilde{\psi }\left(a\xi \right)}e^{i\xi b}d\xi . \end{array}$$ (13)

Finally, the Wiener-Khinchin theorem states that the Fourier transform of the auto-correlation function of a process *x* equal the PSD of the process *x*. Consequently,

$$\begin{array}{c} T_{\Gamma }\left(b,a\right)=\frac{1}{2\pi }\sqrt{a}\int_{-\infty }^{+\infty }\Sigma \left(\xi \right)\bar{\tilde{\psi }\left(a\xi \right)}e^{i\xi b}d\xi . \end{array}$$ (14)

## Supporting information

S1 Fig Boxplots of the sensitivity, precision and F-measure of the detectors ran over this benchmark for different SNRs. The first column is identical to Fig 6 and shows the results for every channels and the other columns represent the result for each channel. The sensitivity of all detectors increases for each channels with the SNR. The STE and HIL has different behaviors for the group of channels I6,PM6, PM10, OT’8 and CR5 compared to B’1, GPH’2 and A’1. The MNI detectors switches into the “no baseline” mode for B’1, GPH’2 and CR5. Delphos and the SLL detectors have consistent behavior across channels.
(TIF)
