# Supplementary figures and images for: What are the assets and weaknesses of HFO detectors? A benchmark framework based on realistic simulations
Source: PLoS One. 2017 Apr 13;12(4):e0174702. doi: 10.1371/journal.pone.0174702 (PMC5390983; doi:10.1371/journal.pone.0174702)

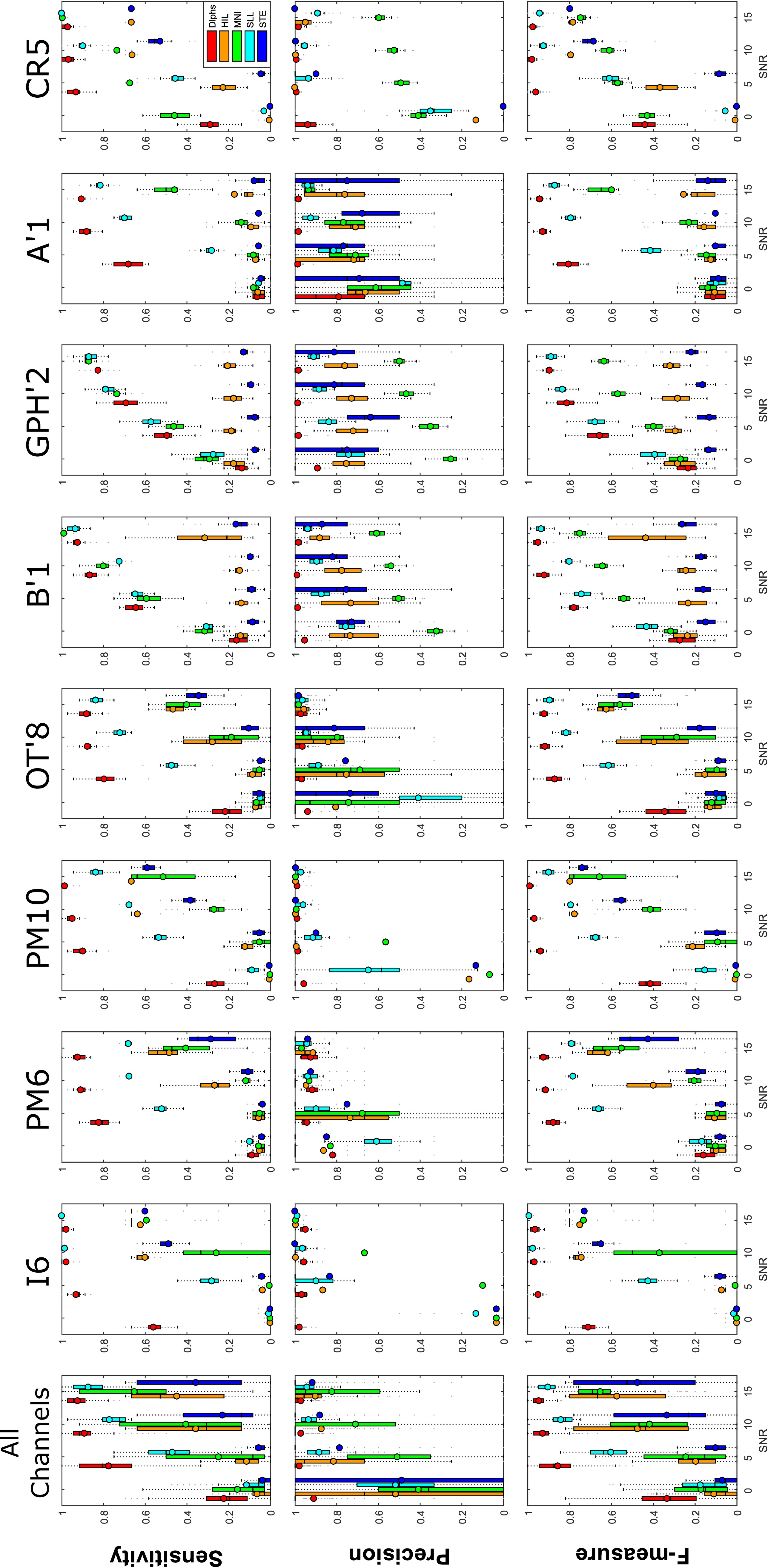

Supplement: S1 Fig — The first column is identical to Fig 6 and shows the results for every channels and the other columns represent the result for each channel. The sensitivity of all detectors increases for each channels with the SNR. The STE and HIL has different behaviors for the group of channels I6,PM6, PM10, OT’8 and CR5 compared to B’1, GPH’2 and A’1. The MNI detectors switches into the “no baseline” mode for B’1, GPH’2 and CR5. Delphos and the SLL detectors have consistent behavior across channels. (TIF) [file pone.0174702.s001.tif]
